# Loss of Tpm4.1 leads to disruption of cell-cell adhesions and invasive behavior in breast epithelial cells via increased Rac1 signaling

**DOI:** 10.18632/oncotarget.16825

**Published:** 2017-04-04

**Authors:** SukYeong Jeong, SunYoung Lim, Galina Schevzov, Peter W. Gunning, David M. Helfman

**Affiliations:** ^1^ Department of Biological Sciences, Korean Advanced Institute of Science and Technology, Daejeon, Republic of Korea; ^2^ Oncology Research Unit, School of Medical Sciences, UNSW Australia, Sydney, NSW, Australia

**Keywords:** tropomyosin, TPM4, migration, invasion, cell adhesions

## Abstract

Here we report the identification and characterization of a novel high molecular weight isoform of tropomyosin, Tpm4.1, expressed from the human *TPM4* gene. Tpm4.1 expression is down-regulated in a subset of breast cancer cells compared with untransformed MCF10A breast epithelial cells and in highly metastatic breast cancer cell lines derived from poorly metastatic MDA-MD-231 cells. In addition, patients with invasive ductal breast carcinoma show decreased *TPM4* expression compared with patients with ductal breast carcinoma *in situ*, and low *TPM4* expression is associated with poor prognosis. Loss of Tpm4.1 using siRNA in MCF10A cells increases cell migration in wound-healing and Boyden chamber assays and invasion out of spheroids as well as disruption of cell-cell adhesions. Down-regulation of Tpm4.1 in MDA-MB-231 cells leads to disruption of actin organization and increased cell invasion and dissemination from spheroids into collagen gels. The down-regulation of Tpm4.1 induces Rac1-mediated alteration of myosin IIB localization, and pharmacologic inhibition of Rac1 or down-regulation of myosin IIB using siRNA inhibits the invasive phenotypes in MCF10A cells. Thus Tpm4.1 plays an important role in blocking invasive behaviors through Rac1-myosin IIB signaling and our findings suggest that decreased expression of Tpm4.1 might play a crucial role during tumor progression.

## INTRODUCTION

Tropomyosins are a family of actin filament binding proteins. Humans contain four tropomyosin genes, *TPM1*, *TPM2*, *TPM3*, and *TPM4*. At least 22 different isoforms are generated from the genes via alternative promoters and alternative RNA splicing. In general tropomyosin isoforms are categorized in two groups, high molecular weight (HMW, 284 amino acids) and low molecular weight (LMW, 248 amino acids) [1]. At the structural level tropomyosins are elongated proteins that possess a dimeric α-helical coiled-coil structure along their entire length. The coiled-coil structure is based on a repeated pattern of seven amino acids with hydrophobic residues at the first and fourth positions and is highly conserved in all tropomyosin isoforms from yeast to human. Although they appear to exhibit a relatively simple protein structure, molecular and genetic studies revealed a level of functional complexity among metazoan tropomyosins. Different tropomyosin isoforms have distinct properties and cellular functions including regulation of contractility, actin filament dynamics, cell motility, intracellular transport and regulation of cell signaling [2–5].

Tropomyosins are among the most studied structural proteins of the actin cytoskeleton that are implicated in alterations of actin filament organization in cellular transformation and cancer. Oncogene-mediated disruption of stress fibers and associated adhesive structures contribute to enhanced motility and invasiveness of tumor cells. Decreased expression of specific nonmuscle tropomyosin isoforms is commonly associated with the transformed phenotypes and down-regulation of HMW tropomyosin isoforms in transformed and cancer cells has been reported in many studies [5–7].

Local invasion, a first step of metastasis, requires of cancer cells to detach from the primary tumor and break through the basement membrane and ECM [8, 9]. In the formation and maintenance of cell-cell adhesions between cells, various actin binding proteins participate by regulating actin cytoskeleton organization [10]. Re-induction of HMW tropomyosin recovers cell-cell adhesion of transformed cells. Migration and invasion of cancer cells are also regulated by HMW tropomyosin. Decreased expression of HMW tropomyosin increases migration of transformed cells and re-induction of HMW tropomyosin in cancer cells decreases migration, invasion, and lung metastasis [11–13].

Virtually all studies of HMW tropomyosins have focused on the products of the *TPM1* and *TPM2* genes. Based on cDNA cloning in ovary tumor tissue and the cervical cancer cell line, HeLa, a HMW tropomyosin isoform is expressed from the human *TPM4* gene [1]. This protein product of the *TPM4* gene is called Tpm4.1 based on a new systematic nomenclature of Tpm isoforms [14]. However, despite the determination of its existence, no subsequent studies have characterized the role of Tpm4.1 in the transformed phenotype.

Here we identify and characterize Tpm4.1 in human breast epithelial cells. We show that Tpm4.1 is down-regulated in various breast cancer cell types. Furthermore, down-regulation of Tpm4.1 induces disruption of cell-cell adhesions and increased migration and invasion in untransformed MCF10A breast epithelial cells and increases invasion in poorly metastatic MDA-MB-231 breast cancer cells. Depletion of Tpm4.1 activates Rac1 and results in redistribution of myosin IIB mediated by Rac1, which are responsible for induction of the invasive phenotypes. Our findings suggest that down-regulation of Tpm4.1 is a critical event during tumor progression that contributes to the metastatic potential of some breast cancer types.

## RESULTS

### The *TPM4* gene expresses a high molecular weight tropomyosin isoform that is down-regulated in breast cancer cells and is associated with invasive breast cancer

**Figure 1:**
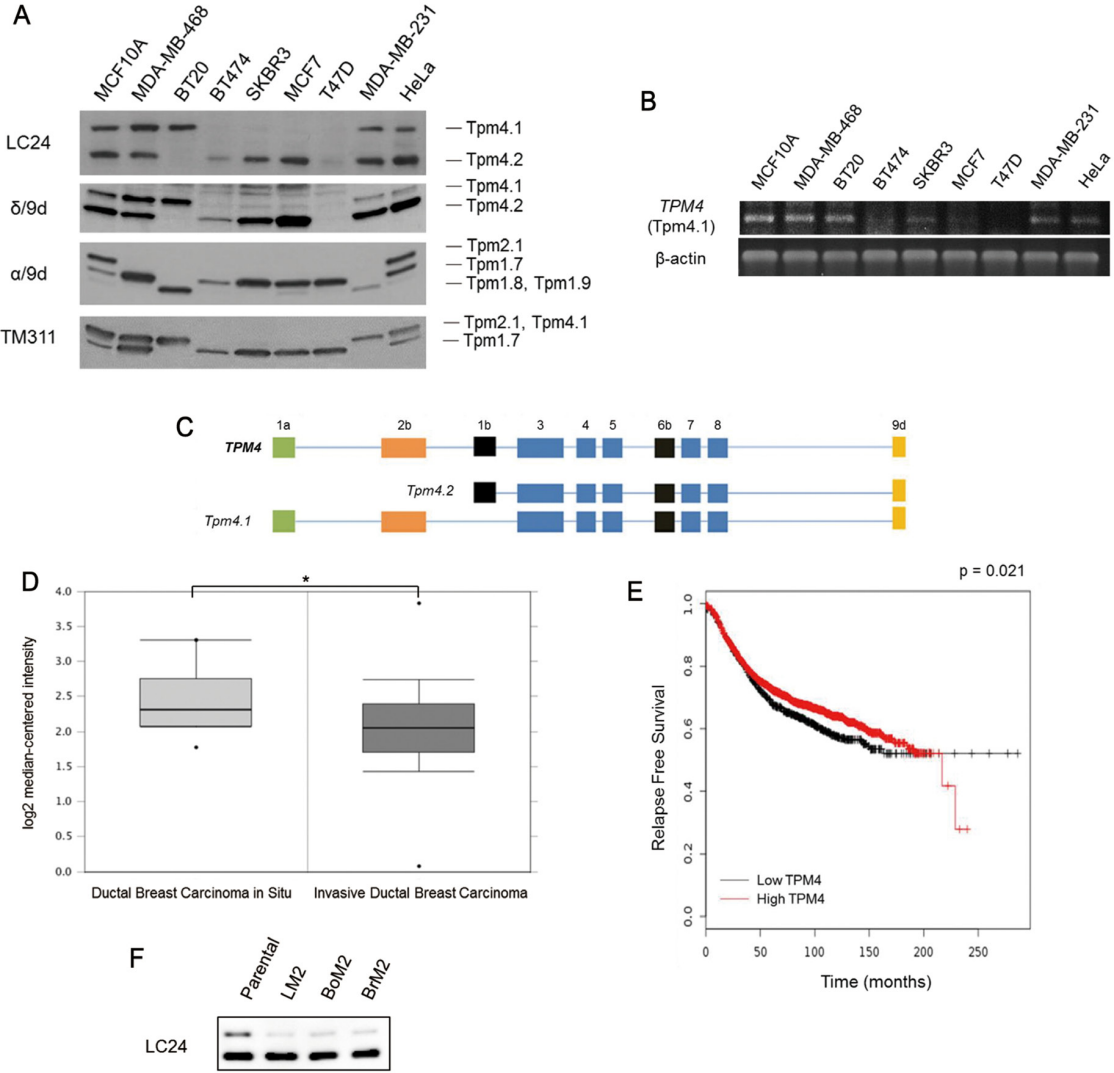
Expression of Tpm4.1 in breast cancer cells (**A**) Detection of tropomyosin isoforms with indicated antibodies in an untransformed breast epithelial cell line, MCF10A, and breast cancer cell lines, MDA-MB-468, BT20, BT474, SKBR3, MCF7, T47D, and MDA-MB-231 by immunoblot. HeLa was used for a standard for the expression of Tpm4.1. (**B**) mRNA expression of Tpm4.1 in a normal breast cell line and breast cancer cell lines was measured by RT-PCR. (**C**) Schematic diagram of the exon organizations of Tpm4.1 and Tpm4.2 expressed from the *TPM4* gene [5]. (**D**) Oncomine box plot of *TPM4* expression levels in DCIS (*n* = 10) and IDC (*n* = 1,556) [38]. *p < 0.05. (**E**) Kaplan-Meier curve of relapse free survival of breast cancer patients with a compilation of all breast cancer patients independent of subcategories (*n* = 3,554). Data was obtained from the Kaplan-Meier plotter breast cancer database [39]. (**F**) Endogenous expressions of Tpm4.1 and Tpm4.2 in poorly metastatic parental MDA-MB-231 and its derivative highly metastatic cell lines, LM2, BoM2, and BrM2.

Previous studies of HMW tropomyosins in breast and other cancers have focused exclusively on the gene products of the *TPM1* and *TPM2* genes. This is because the occurrence of HMW tropomyosin isoforms from the *TPM3* or *TPM4* genes has been unexplored. In experiments comparing the expression of tropomyosins in various human breast cancer cell lines with untransformed MCF10A breast epithelial cells we observed that the LC24 antibody that was raised against sequences in the carboxy-terminal domain of the *TPM4* gene detected the well-characterized LMW Tpm4 isoform, Tpm4.2, but also a protein with the same mobility as a HMW tropomyosin (Figure 1A). Previous immunoblot studies have suggested that the LC24 antibody cross-reacts with Tpm2.1, a HMW tropomyosin isoform encoded by the *TPM2* gene, and a HMW tropomyosin band detected by LC24 is Tpm2.1 [15, 16]. To further analyze the expression of this HMW tropomyosin and to identify what the HMW tropomyosin is, another antibody, α/9d, that reacts against sequences in the C-terminal domain of Tpm2.1 (*TPM2* gene product) and Tpm1.6 and Tpm1.7 (*TPM1* gene products) was used. Curiously, although immunoblot analysis using the LC24 antibody detected a band corresponding to a HMW tropomyosin in MCF10A, MDA-MB-468, BT-20, MDA-MB-231 and HeLa cells, the α/9d antibody only detected a corresponding band in MCF10A and HeLa cells but not in the other cell lines. Identical results to the LC24 antibody were obtained using the δ/9d polyclonal antibody, which recognizes sequences in the C-terminal domain of Tpm4 isoforms. Furthermore, using the TM311 antibody that recognizes sequences in the N-terminal domain of HMW tropomyosins also detected a HMW tropomyosin expressed in MCF10A, MDA-MB-468, BT-20, MDA-MB-231, and HeLa cells that corresponded in position to the HMW tropomyosin. With the immunoblot results of tropomyosin antibodies, we concluded the detected HMW tropomyosin isoform as Tpm4.1. The siRNA sequences designed to specifically silence Tpm4.1 also depleted only Tpm4.1 but not Tpm2.1 (Figure 3A). These results show that Tpm4.1 is a distinct tropomyosin isoform and its detection is not the result of cross-reaction with other TPM gene products.

Prompted by our immunoblot data, we carried out experiments to further confirm the altered expression levels of Tpm4.1 in breast cancer cells. RNA was isolated from the various breast cancer cell lines and subjected to RT-PCR (Figure 1B). In agreement with the immunoblot analysis, mRNA corresponding to Tpm4.1 was detected strongly in MCF10A, MDA-MB-468, BT-20, MDA-MB-231 cells and HeLa cells but not in the other cell lines. The down-regulation of Tpm4.1 mRNA and protein in breast cancer cells suggests that Tpm4.1 is related to breast cancer progression.

To investigate the relationship between progression of breast cancer and tropomyosin isoforms, we interrogated the expression of *TPM4* in breast cancer patients. Patients with invasive ductal breast carcinoma showed decreased *TPM4* expression compared with patients with ductal breast carcinoma *in situ* (Figure 1D). In addition, low *TPM4* expression is associated with poor prognosis in breast cancer patients (Figure 1E). We further evaluated the expression of Tpm4.1 and Tpm4.2 in metastatic models of MDA-MB-231 cells that have a preference for metastasis to lung (LM2), bone (BoM2) or brain (BrM2) (Figure 1F) [17–19]. By comparison to poorly metastatic parental MDA-MB-231 cells, the three highly metastatic cell lines LM2, BoM2, and BrM2 showed decreased expression of Tpm4.1 but not of Tpm4.2. Collectively these experiments suggest that down-regulation of Tpm4.1 might contribute to invasive and metastatic characteristics in breast cancer.

### Decreased expression of Tpm4.1 increases the rate of cell migration and invasion

**Figure 2:**
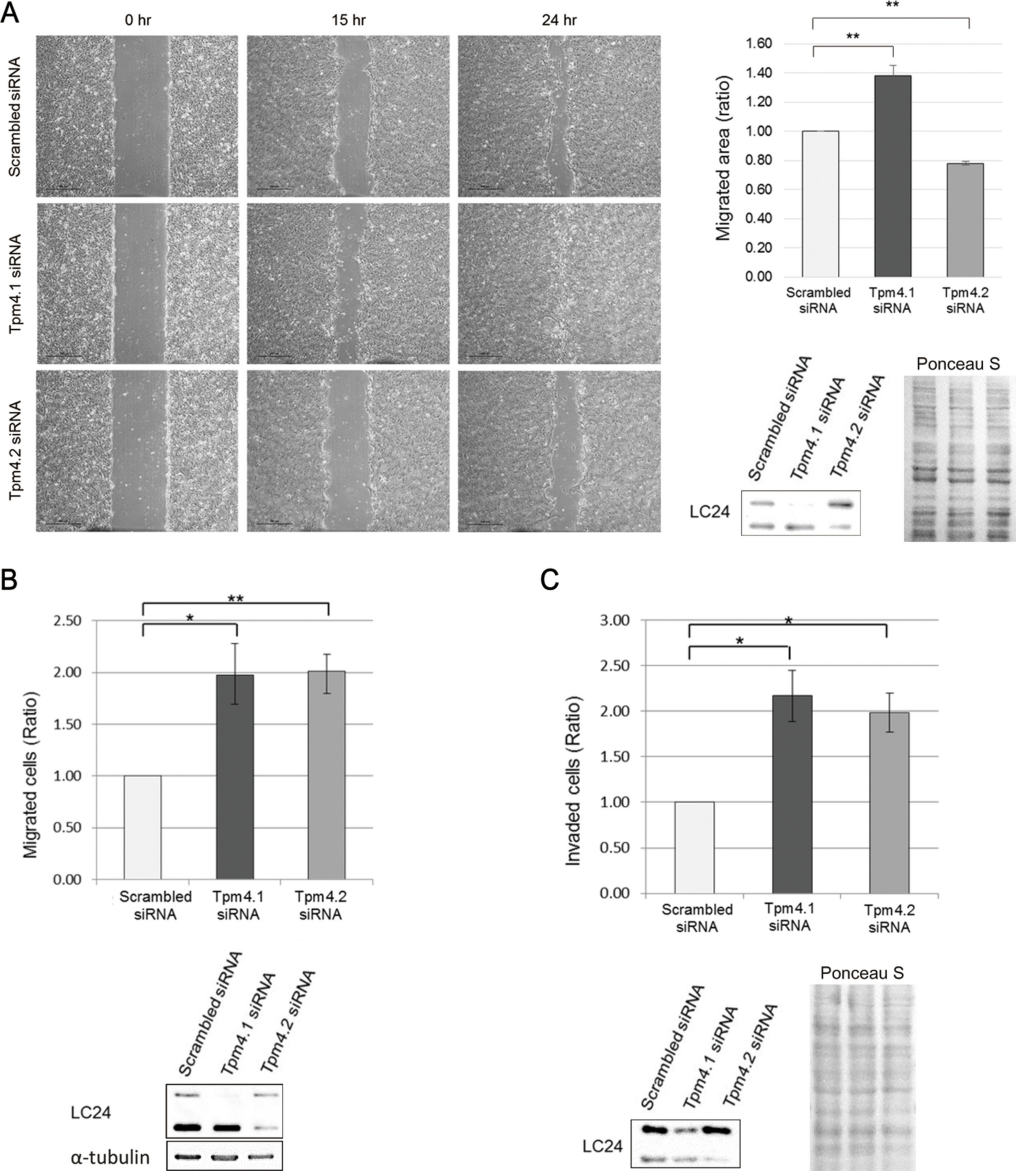
Loss of Tpm4 isoforms affects cell migration and invasion (**A**) Migratory ability of MCF10A cells with siRNAs was analyzed by wound healing assay. Left panel: Representative images of wound healing assay. Scale bars, 500 μm. Right upper panel: The graph was from three independent experiments. *p < 0.05, **p < 0.005. Right lower panel: Representative images of immunoblot. (**B**–**C**) Upper panel: Analysis of migration (B) and invasion (C) of MCF10A cells with the indicated siRNAs in Boyden chamber assay with uncoated and Matrigel-coated membrane respectively. Each graph was from three independent experiments. *p < 0.05, **p < 0.005. Lower panel: Representative images of immunoblot.

The down-regulation of Tpm4.1 in various breast cancer cells compared to untransformed MCF10A cells suggests that the loss of Tpm4.1 could play a causal role in tumor progression. Because two isoforms are expressed from the *TPM4* gene: Tpm4.1 and Tpm4.2 (Figure 1C), we asked if down-regulation of either Tpm4.1 or Tpm4.2 in MCF10A cells affects cell migration and invasion. We developed siRNAs against Tpm4.1 and compared their effects with siRNA against Tpm4.2. The depletion of Tpm4.1 by the pool of Tpm4.1 siRNAs resulted in a faster migration of MCF10A cells in wound-healing assay compared to control cells (Figure 2A).

**Figure 3:**
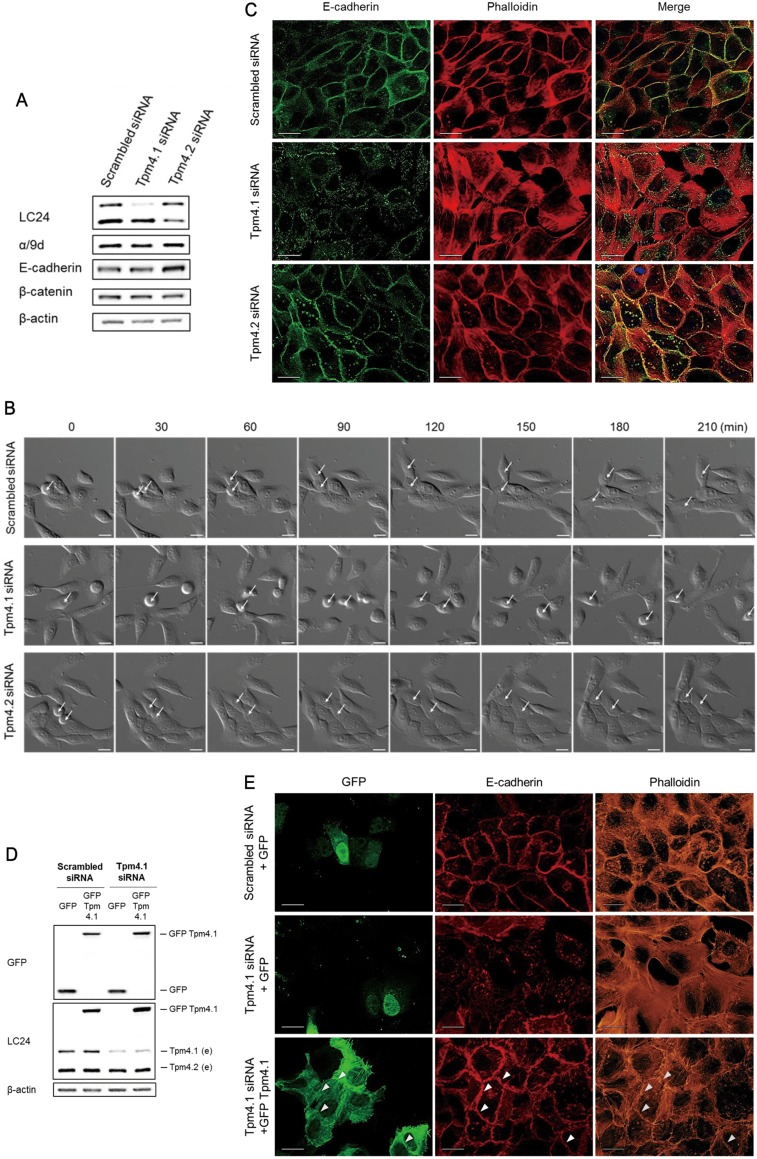
Loss of Tpm4.1 impairs cell-cell adhesions (**A**) Expressions of tropomyosin isoforms and cell-cell junction components were analyzed by immunoblot after siRNA transfection. (**B**) Time-lapse images of MCF10A cells with the indicated siRNAs in every 30 minutes. Arrows, dividing cells. Scale bars, 20 μm. (**C**) MCF10A cells with the indicated siRNAs were stained with the E-cadherin antibody and phalloidin to detect F-actin. Scale bars, 20 μm. (**D**) Rescue of cell-cell junctions by cDNA transfection following siRNA transfection. Immunoblot analysis was done after siRNA and cDNA plasmids transfection (e, endogenous tropomyosin isoforms). (**E**) MCF10A cells with the indicated siRNAs and cDNA plasmids were stained with the E-cadherin antibody and phalloidin to detect F-actin. Arrowheads indicate E-cadherin and actin bundles along cell-cell adhesions among the GFP-Tpm4.1-transfected cells. Scale bars, 20 μm.

We then performed Boyden chamber assays. Tpm4.1 depletion in MCF10A cells using siRNA increased migration and invasion in both naked PET (polyethylene terephthalate) membrane and membrane coated with a three-dimensional Matrigel matrix respectively (Figure 2B and 2C). The migration and invasion assays show that loss of Tpm4.1 can contribute to increased cell migration and invasion. By comparison, with Tpm4.2 depletion, cell migration decreased in wound healing assay but increased in Boyden migration assay, and cell invasion increased (Figure 2A–2C).

### Depletion of Tpm4.1 impairs cell-cell adhesions and alters the actin cytoskeleton

Enhancement in cell migration is associated with impaired cell-cell junctions [20, 21]. In addition, loss of cell-cell adhesions is an essential step for local invasion and metastasis [9]. To learn more about how Tpm4.1 depletion affects cell motility we examined the organization of the actin cytoskeleton and cell-cell adhesions following down-regulation of Tpm4.1. First we asked if down-regulation of Tpm4.1 or Tpm4.2 in MCF10A cells effected cell-cell adhesions. When MCF10A cells treated with scrambled siRNA were seeded on laminin, the divided cells attached to neighboring cells and stably maintained the attachment to other cells in a colony (Figure 3B and Supplementary Movie 1). Tpm4.1 depletion resulted in unstable cell-cell adhesions among neighboring cells and the cells exhibited a decreased ability to form a colony with other cells because of frequent detachment (Figure 3B and Supplementary Movie 2). By contrast depletion of Tpm4.2 did not result in perturbed cell-cell adhesions and the cells formed a more stably maintained colony compared with control (Figure 3B and Supplementary Movie 3).

We next examined the effects of Tpm4.1 in cell-cell junctions. Consistent with unstable cell-cell adhesions, Tpm4.1 depletion impaired E-cadherin localization. In addition, loss of Tpm4.1 was accompanied by disruption of well-organized cortical actin bundles along cell-cell adhesions and enhanced formation of stress fibers (Figure 3C). The transfection of single Tpm4.1 siRNAs also exhibited impairment of cell-cell adhesions (Supplementary Figure 1B). By contrast, depletion of Tpm4.2 did not induce disorganization of E-cadherin and actin bundles but still maintained accumulation of E-cadherin at cell-cell adhesions and even increased E-cadherin expression (Figure 3A and 3C). To confirm the effects of Tpm4.1 depletion, we carried out a rescue experiment. We transfected Tpm4.1 cDNA plasmid with silent mutations in the siRNA-targeted sequences into the Tpm4.1-silenced cells by siRNA (Figure 3D). The rescue experiment showed the recovery of cell-cell adhesions with accumulation of E-cadherin and well-organized cortical actin bundles (Figure 3E). Together, these results indicate that loss of Tpm4.1 is involved in decreased cell-cell adhesions.

### Depletion of Tpm4.1 induces morphological changes in MDA-MB-231

**Figure 4:**
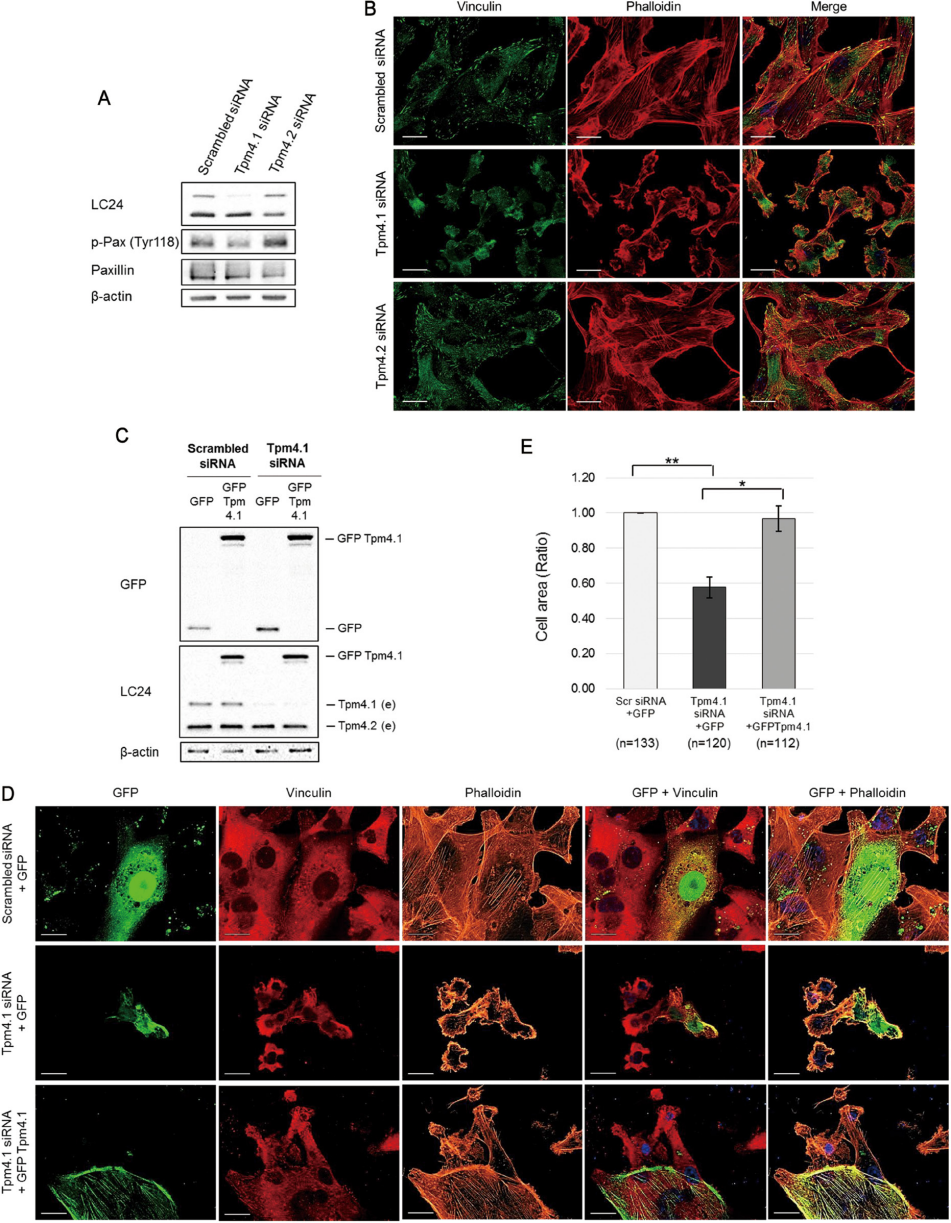
Loss of Tpm4.1 decreases stress fibers and focal adhesions in MDA-MB-231 (**A**) MDA-MB-231 cell lysates were analyzed by immunoblot after siRNA transfection to examine silencing efficiency and phosphorylation of paxillin. (**B**) MDA-MB-231 cells with the indicated siRNAs were stained with the vinculin antibody and phalloidin. Scale bars, 20 μm. (**C**) Immunoblot analysis after siRNA and cDNA plasmids transfection (e, endogenous tropomyosin isoforms). (**D**) MDA-MB-231 cells with the indicated siRNAs and cDNA plasmids were stained with the vinculin antibody and phalloidin. (**E**) The cDNA plasmid-transfected cells were randomly captured and the sizes of the cells were measured using ImageJ software. The cell sizes were averaged as division of the measured area by the number of nucleus. n, nucleus. The graph was from three independent experiments. *p < 0.05, **p < 0.005

Our immunoblot analysis showed reduced expression of Tpm4.1 in highly metastatic cell lines, LM2, BoM2 and BrM2 compared to poorly metastatic MDA-MB-231 cells (parental) from which they were derived (Figure 1F). LM2 is a lung metastatic cell line, which shows enhanced migratory and invasive characteristics compared with the parental MDA-MB-231 cells. In addition, a previous study revealed that parental cells had well-developed stress fibers whereas LM2 cells had poorly organized stress fibers [22]. To examine whether decreased expression of Tpm4.1 is involved in these phenotypic differences between parental and LM2 cells, we silenced Tpm4.1 in parental MDA-MB-231 cells. Depletion of Tpm4.1 reduced stress fiber and resulted in a transition from spread to rounded cell morphology (Figure 4B). Down-regulation of Tpm4.1 also resulted in loss of focal adhesions detected by vinculin antibody staining and reduced phosphorylation of paxillin at Tyr 118, which are the components of focal adhesions (Figure 4A and 4B). These alterations in the actin cytoskeleton following Tpm4.1 depletion in MDA-MB-231 cells is similar with the morphological differences between parental and LM2 cells as the previous study reported. To confirm the effects of Tpm4.1 depletion, we carried out a rescue experiment. The transfection of the Tpm4.1 cDNA plasmid with silent mutations in Tpm4.1-depleted cells recovered stress fibers and cell spreading (Figure 4C–4E). Thus, Tpm4.1 plays a critical role in the organization of actin filaments.

### Localization of Tpm4.1 in actin structures and cell-cell junctions in MCF10A cells

**Figure 5:**
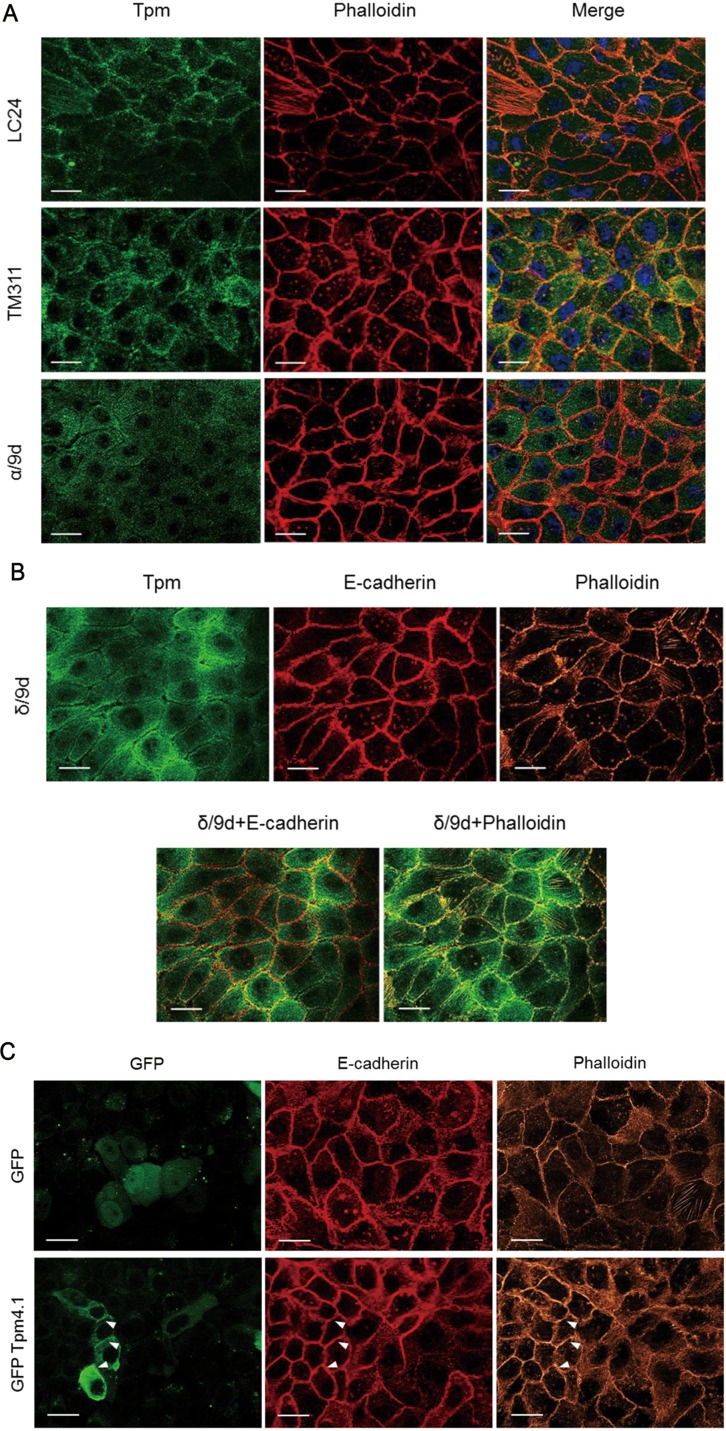
Localization of tropomyosin in MCF10A cells (**A**) MCF10A cells in confluent condition were stained with the indicated tropomyosin antibody and phalloidin. Scale bars, 20 μm. (**B**) MCF10A cells were stained with δ/9d and E-cadherin antibodies and phalloidin. Scale bars, 20 μm. (**C**) After transfection of GFP and GFP-Tpm4.1, MCF10A cells were stained with E-cadherin antibody and phalloidin. Scale bars, 20 μm.

Changes in the organization of the actin cytoskeleton in transformed cells are associated with cell migration, invasion and alterations in cell-cell junctions. Our findings showed that down-regulation of Tpm4.1 using siRNA altered cell morphology, actin structures and cell adhesions in MCF10A and MDA-MB-231. To further investigate the role of Tpm4.1 in cell-cell adhesions, we examined the localization of tropomyosin in confluent cell monolayers with cell-cell junctions in MCF10A cells. Staining with mAb LC24, which recognizes Tpm4.1 and Tpm 4.2 isoforms, showed co-localization in the cell periphery with cortical actin bundles (Figure 5A). Staining with mAb TM311 that recognizes only HMW tropomyosins also showed co-localization with cortical actin bundles. Staining with the polyclonal δ/9d antibody, which recognizes both Tpm4 isoforms, co-localized with E-cadherin and cortical actin bundles (Figure 5B). However, the α/9d antibody, which does not recognize *TPM4* gene products showed puncta at cortical regions but the detection was lower compared with the other three tropomyosin antibodies (Figure 5A). To further examine the localization of Tpm4.1 we transfected plasmids encoding GFP-Tpm4.1. The transfection of GFP-tagged Tpm4.1 showed co-localization with E-cadherin and cortical actin bundles in agreement with the antibody staining results (Figure 5C). The localization of Tpm4.1 with E-cadherin and cortical actin bundles along cell-cell adhesions further suggests that Tpm4.1 functions in stable cell-cell adhesions.

### Loss of Tpm4.1 affects spheroid formation and invasion

**Figure 6:**
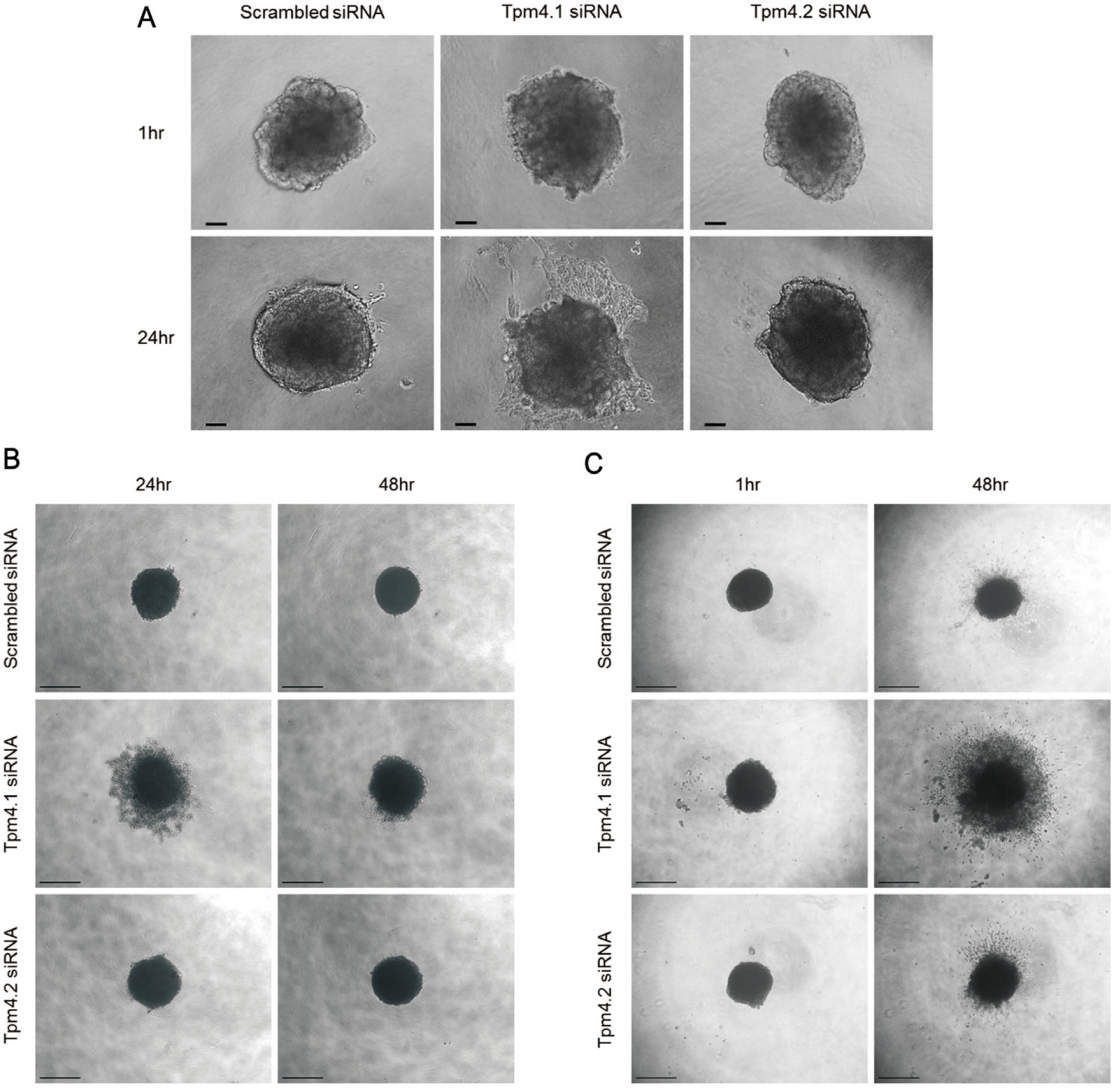
Tpm4.1 regulates cell spheroid aggregation and invasion (**A**) Representative images of MCF10A spheroids with the indicated siRNA embedded in collagen I. Scale bars, 100 μm. (**B**) MDA-MB-231 cells with the indicated siRNA were grown in ultra-low attachment plate with Matrigel to form spheroids. Scale bars, 500 μm. (**C**) Spheroids of MDA-MB-231 cells were embedded in collagen I. Scale bars, 500 μm.

The experiments above demonstrated that Tpm4.1 disrupted cell-cell adhesions and increased cell migration and invasion, which are important properties of cancer cells to facilitate local invasion. We extended our studies using 2D culture conditions in a 3D culture condition to examine whether Tpm4.1 depletion also stimulates invasion under *in vivo*-like condition. 3D multicellular tumor spheroid system mimics the primary tumor architecture with similar morphological and physiological characteristics, and embedding of multicellular spheroids in type I collagen is a proper model to examine invasion of cells into the surrounding matrix [23, 24]. Spheroids of MC10A cells were formed and then embedded in type I collagen gel. Tpm4.1 depletion increased invasion out of spheroids, similar to what was observed in single cell invasion in Boyden invasion assay (Figure 6A). The invasion following Tpm4.2 depletion was not significant (Figure 6A). Next we examined the role of Tpm4.1 and Tpm4.2 in spheroid formation and invasion in MDA-MB-231 cells. As noted above, Tpm4.1 is down-regulated in highly metastatic cell lines compared to parental MDA-MB-231 cells from which they were derived (Figure 1F), and we asked whether depletions of the Tpm4 isoforms make the poorly metastatic parental MDA-MB-231 cells more invasive. Down-regulation of Tpm4.1 using siRNA resulted in slower aggregation of spheroids in MDA-MB-231 cells (Figure 6B). The spheroids composed of Tpm4.1-depleted cells exhibited increased cell invasion and dissemination from spheroid into collagen gels similar to what we observed in spheroids of MCF10A cells (Figure 6C). By contrast, Tpm4.2 depletion gave insignificant effects on spheroid invasion. These results show that depletion of Tpm4.1 stimulates cells to be invasive in 2D and 3D culture conditions in both MCF10A and MDA-MB-231 cells, which suggests down-regulation of Tpm4.1 may play a role in enhanced invasive behaviors of cancer cells during metastasis.

### Tpm4.1 is involved in regulation of Rac1 activity and its function in cell migration

**Figure 7:**
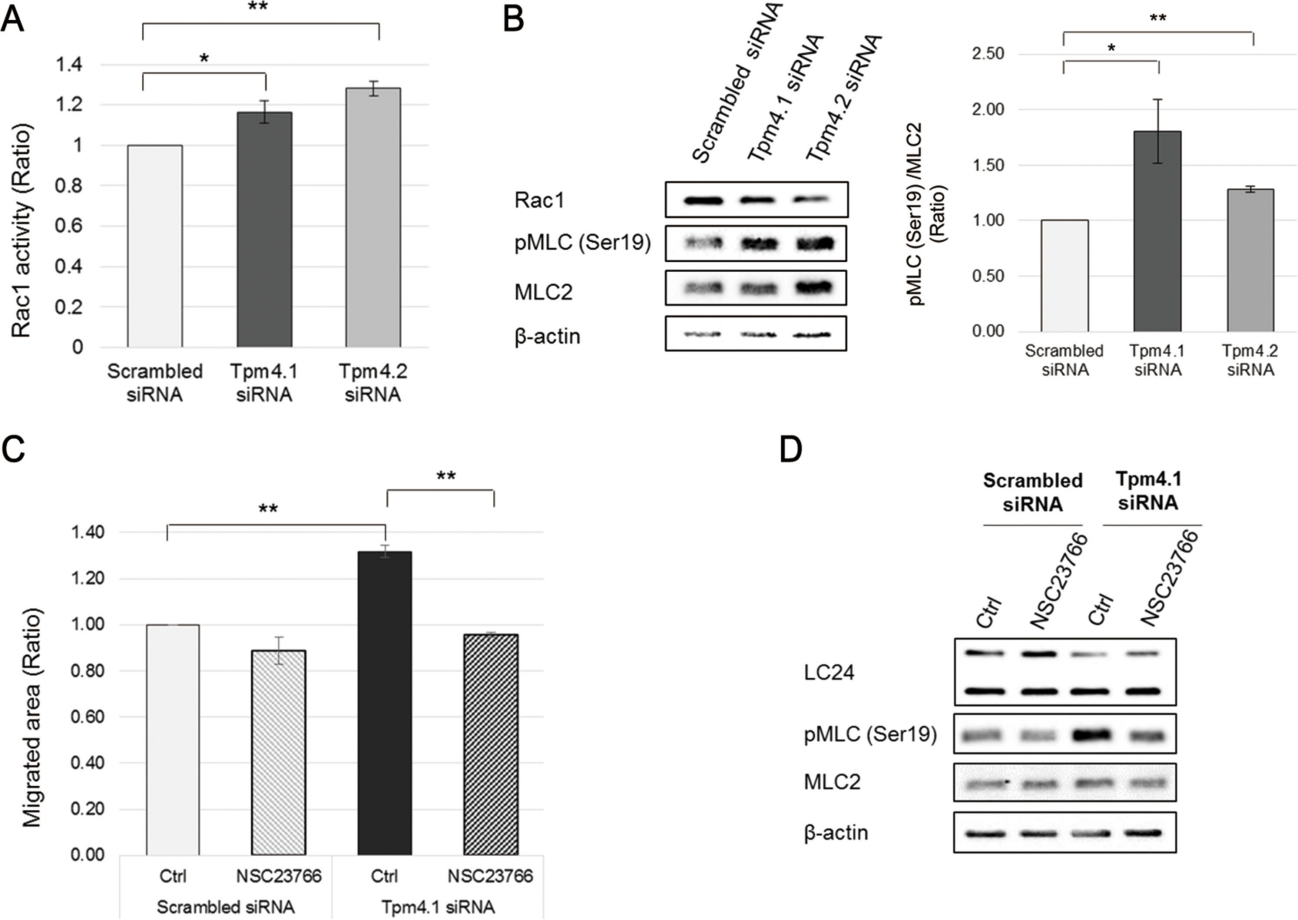
Depletion of Tpm4.1 induces Rac1 activation and it is responsible for increase in cell migration (**A**) The level of GTP-bound Rac1 was measured by G-LISA Rac1 activity assay in MCF10A cells after siRNA transfection. The graph was from three independent experiments. *p < 0.05, **p < 0.005 (**B**) Left panel: Immunoblot analysis of Rac1 activity assay samples. Right upper panel: The intensity of p-MLC band was divided by that of total MLC band. The graph was from the samples of three independent experiments used in the analysis. *p < 0.05, **p < 0.005. (**C**) siRNA-transfected MCF10A cells were treated with 50 μM NSC23766 and migratory ability was analyzed by wound healing assay. The graph was from three independent experiments. *p < 0.05, **p < 0.005 (**D**) Immunoblot analysis of wound healing assay samples.

We next investigated how loss of Tpm4.1 induces the invasive behaviors. Depletion of Tpm4.1 changed cell morphologies with reorganized actin structures in different cell lines (Figures 3 and 4). These results suggested a possibility that the invasive behavior is derived from changes in actin organization. The importance of actin structures in regulation of cell polarity for cell-cell adhesions, cell migration and invasion has been demonstrated in many papers [10, 25]. Tropomyosins can regulate activity of myosin II and thereby affects the organization of actin structures [3, 26]. To examine a possibility that tropomyosin-dependent actin organization is involved in the induction of the invasive behaviors, we first analyzed the activity of Rac1 following Tpm4.1 silencing. Rac1, a member of the Rho GTPase family, regulates cell morphology, migration, invasion and cell-cell adhesions with altered actin organization and actomyosin contractility [27–29]. Depletion of Tpm4.1 increased the activity of Rac1 (Figure 7A). Inhibition of Rac1 using a Rac1-specific inhibitor, NSC23766, rescued the increased cell migration following Tpm4.1 depletion (Figure 7C). These results show that Rac1 is involved in the regulation of invasive behaviors by Tpm4.1.

We also checked two well-known proteins that regulate cell-cell adhesions and cell migration through organization of actin structures and actomyosin contractility, Myosin light chain kinase (MLCK) and Rho-associated coiled-coil-containing protein kinase (ROCK) [30–33]. However, the MLCK inhibitor attenuated ordinary migration of MCF10A cells while the ROCK inhibitor did not revert the increased migration following Tpm4.1 depletion (Supplementary Figures 2B and 3B), which implies that MLCK and ROCK are not involved in cell migration regulated by Tpm4.1.

### Redistribution of myosin IIB mediated by activated Rac1 after Tpm4.1 silencing increases cell migration and disrupts cell-cell adhesions

**Figure 8:**
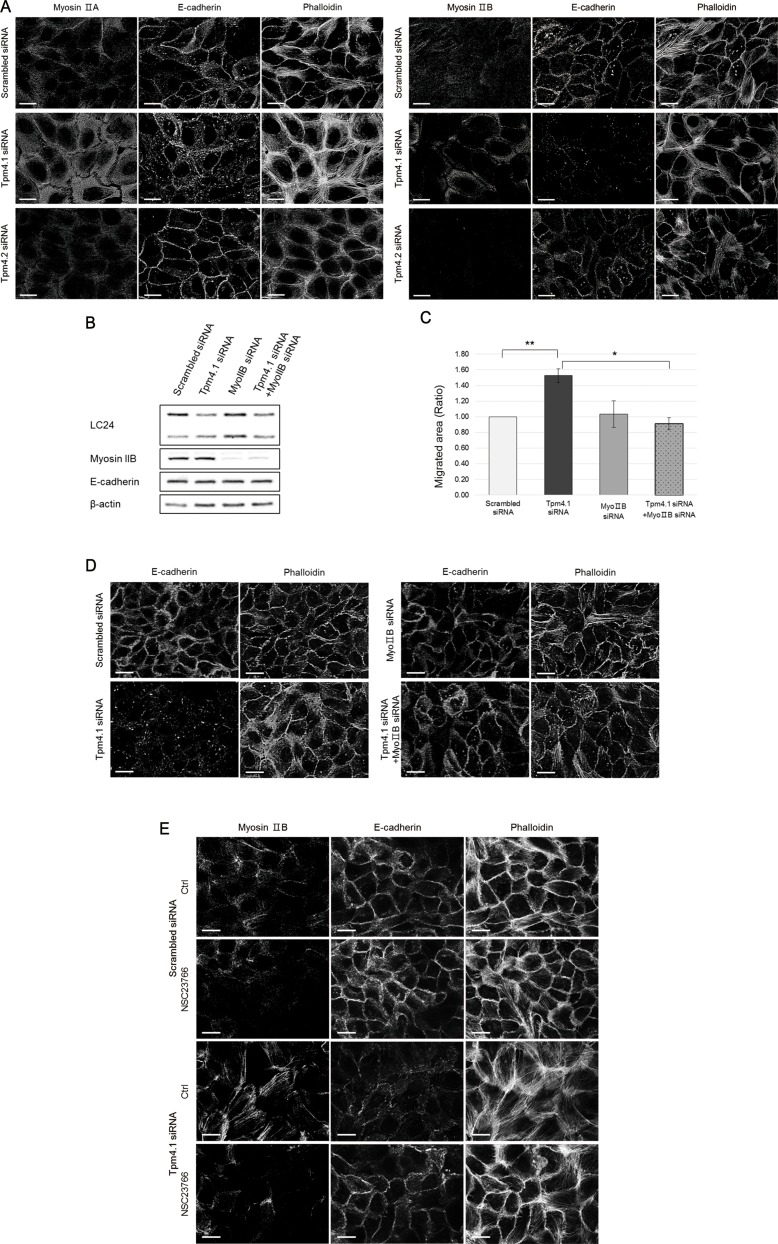
The Rac1 activation induces redistribution of myosin IIB and it induces the invasive phenotypes of Tpm4.1-silenced cells (**A**) Immunofluorescence localization of myosin IIA and myosin IIB in MCF10A cells with the indicated siRNAs. Scale bars, 20 μm. (**B**) Immunoblot analysis after siRNA transfection. (**C**) Migratory ability of MCF10A cells with the indicated siRNAs was analyzed by wound healing assay. The graph was from three independent experiments. *p < 0.05, **p < 0.005 (**D**) MCF10A cells with the indicated siRNAs were stained with the E-cadherin antibody and phalloidin. Scale bars, 20 μm. (**E**) Immunofluorescence localization of myosin IIB with treatment of 50 μM NSC23766 for 1 hr in MCF10A cells with the indicated siRNAs.

Rac1 regulates actin reorganization and actomyosin contractility. We next investigated the involvement of myosin II in the phenotypes following Tpm4.1 silencing. Because myosin II is important in the stability of cell-cell junctions, we examined whether depletion of Tpm4.1 disrupts cell-cell adhesions through myosin II. First we examined the distribution of the two isoforms of myosin II expressed in MCF10A cells, myosin IIA and IIB. Tpm4.1 silencing induced alterations of myosin distribution that myosin IIA and IIB became associated with stress fibers (Figure 8A). However, in contrast with Tpm4.1 silencing, cells transfected with Tpm4.2 siRNA did not show any difference in distribution of both myosin IIA and IIB (Figure 8A).

To determine whether the alteration of cell migration by active Rac1 is dependent on a specific myosin II isoform, we transfected myosin IIA or IIB siRNAs respectively together with Tpm4.1 siRNA. Silencing myosin IIB inhibited the increased cell migration following Tpm4.1 depletion (Figure 8C). Next, we examined whether myosin IIA and IIB play a role in the alterations of cell-cell junctions following depletion of Tpm4.1 because the cells with disrupted cell-cell junctions showed changes in the distribution of myosin IIA and IIB. Silencing myosin IIB recovered cell-cell junctions with accumulation of E-cadherin between contacted cells and cortical actin bundles in Tpm4.1-depleted cells (Figure 8D). In contrast with myosin IIB silencing, transfection of myosin IIA siRNA did not rescue the altered migration and even disrupted cell-cell junctions (Supplementary Figure 4B and 4C).

These results with inhibition of Rac1 and myosin IIB silencing in cell migration and cell-cell junctions suggest a functional relationship between Rac1 and myosin IIB. To clarify the relationship, we analyzed the localization of myosin IIB when cells were treated with the Rac1 inhibitor. The altered distribution of myosin IIB to stress fibers following depletion of Tpm4.1 were recovered with Rac1 inhibition (Figure 8E). Together, these results imply that Rac1 activation regulates distribution of myosin IIB and it plays a critical role in the disruption of cell-cell adhesions in Tpm4.1-depleted cells.

## DISCUSSION

Here we study the role of a previously uncharacterized high molecular weight isoform of tropomyosin Tpm4.1, expressed from the *TPM4* gene, in untransformed and transformed breast epithelial cells. Tpm4.1 expression is down-regulated in some breast cancer cell lines compared to untransformed breast epithelial cells and in highly metastatic breast cancer cell lines compared to poorly metastatic cells (Figure 1). In addition, *TPM4* expression is decreased in invasive ductal breast carcinoma compared with ductal breast carcinoma *in situ* and the low level of expression is associated with a poor prognosis. The decreased expression of Tpm4.1 in breast cancer suggests that loss of this isoform is associated with tumor progression.

To invade into the surrounding environment, cancer cells need to be detached from the primary tumor, which requires disruption of cell-cell adhesions. During the formation and maintenance of cell-cell junctions, actin reorganization is important together with cadherin clustering [34]. Loss of Tpm4.1 disrupts cuboidal cell morphology and actin organization in cell-cell junctions (Figure 3). The cells after transfection of siRNA against Tpm4.1 are unable to maintain new cell-cell contacts (Figure 3B and Supplementary Movie 2). In addition, Tpm4.1 is localized along the portions of cell-cell adhesions with E-cadherin and actin bundles (Figure 5). These observations imply that Tpm4.1 is required for stable cell-cell junctions. In stark contrast with Tpm4.1, the depletion of Tpm4.2 exhibits stable cell-cell adhesions and results in an increase of E-cadherin expression (Figure 3 and Supplementary Movie 3).

Our analysis shows that depletion of Tpm4.1 increases cell migration and penetration of Matrigel which has a similar composition of the basement membrane (Figure 2). This suggests that down-regulation of Tpm4.1 contributes to the ability of cancer cells to breach the basement membrane and then to migrate into the surrounding environment. To extend these results into the similar environment of primary tumors *in vivo*, we used the 3D multicellular tumor spheroid system to form primary tumor-like structures and embedded spheroids into collagen gels to mimic the ECM surrounding the primary tumor. During spheroid formation, in contrast to the aggregation in MCF10A cells, down-regulation of Tpm4.1 in MDA-MB-231 cells induces slower aggregation of cells (Figure 6B). The formation of multicellular spheroids are mediated by cell-cell and cell-matrix interactions [35]. MDA-MB-231 cells, which lose cell-cell adhesions, form spheroids only with the reconstituted basement membrane-like matrix, Matrigel, and when interaction with a matrix is inhibited following depletion of integrin β1 the cells can not form spheroids [35, 36]. This suggests that in MDAMB-231 cells the slower spheroid formation is induced by decreased cell-matrix interactions following Tpm4.1 depletion, as the cells shows decreased formation of focal adhesions (Figure 4). The spheroids composed of Tpm4.1-depleted cells show increased invasion into collagen I in two cell lines, MCF10A and MDA-MB-231 (Figure 6A and 6C). Thus, the results in Matrigel-coated Boyden chambers and in spheroid invasion assay (Figures 2C and 6), show that loss of Tpm4.1 could contribute to local invasion into the basement membrane and the ECM. In contrast to Tpm4.1 depletion, Tpm4.2 depletion shows inconsistent results in invasion. Depletion of Tpm4.2 increases cell invasion in Boyden chamber assay but insignificantly in spheroid invasion (Figures 2C and 6). Actomyosin contractility regulates cell migration and cells in various matrix stiffness conditions require different actomyosin contractility to efficiently migrate into the ECM [37], which would give a hint about the different effects of Tpm4.2 silencing in the 2D and 3D invasion assays, with the no changes in the distribution of myosin IIA and IIB (Figure 8A and 8B).

Depletion of Tpm4.1 induces increased cell migration and invasion and perturbation of cell-cell adhesions. Our experiments to determine how Tpm4.1 is involved in the invasive behaviors show that depletion of Tpm4.1 is accompanied by increased activation of Rac1 (Figure 7A). Inhibition of Rac1 reverts the increased cell migration following Tpm4.1 silencing, which demonstrates the involvement of Rac1 activation regulated by Tpm4.1 (Figure 7C). In addition, depletion of Tpm4.1 induces alterations in the distribution of both myosin IIA and IIB. No differences in the expression levels of myosin IIA and IIB after transfection of Tpm4.1 siRNA imply that the changes induced by depletion of Tpm4.1 are the result of their altered localization (Figure 8B and Supplementary Figure 4A). In spite of the altered distribution of both myosin II isoforms, only depletion of myosin IIB prevents the increase in cell migration and disruption of cell-cell junctions following Tpm4.1 silencing (Figure 8C and 8D). In contrast with myosin IIB, depletion of myosin IIA does not rescue the altered cell migration following Tpm4.1 silencing and even induces disruption of cell-cell junctions (Supplementary Figure 4). These results indicate that the effects of Tpm4.1 on cell migration and cell-cell adhesions are mediated by myosin IIB but not by myosin IIA. Rac1 inhibition in the cells transfected with Tpm4.1 siRNA shows stabilized cell-cell junctions with rescued distribution of myosin IIB (Figure 8E). This demonstrates that the disruption of cell-cell junctions following Tpm4.1 depletion is induced by Rac1-mediated myosin IIB redistribution. It is not clear how activated Rac1 following Tpm4.1 depletion regulates myosin IIB. The activity of myosin II is regulated by phosphorylation of myosin light chains and myosin heavy chains [30]. Depletion of Tpm4.1 shows increase in phosphorylation of myosin light chains which is rescued by Rac1 inhibition (Figure 7D). However, inhibition of other kinases, which also phosphorylate myosin light chains, MLCK and ROCK, do not rescue the increased migration following Tpm4.1 depletion (Supplementary Figures 2 and 3). This suggests that the altered distribution of myosin IIB through Rac1 following Tpm4.1 depletion might not be dependent on phosphorylation of myosin light chains but on other mechanisms such as phosphorylation of myosin IIB heavy chains. Tpm4.2 depletion also increases Rac1 activity and phosphorylation of myosin light chains (Figure 7A and 7B). The expression of myosin light chain 2 is increased slightly following Tpm4.1 silencing and significantly following Tpm4.2 silencing (Figure 7B). In contrast with Tpm4.1 siRNA, Tpm4.2 siRNA does not change the distribution of myosin IIA and IIB (Figure 8A and 8B), which might be responsible for the maintenance of stable cell-cell adhesions following Tpm4.2 silencing. This difference suggests that although down-regulation of Tpm4.1 or Tpm4.2 increases Rac1 activity, each tropomyosin isoform is involved in regulating different downstream effects of Rac1 in cell-cell adhesions, and cell migration and invasion.

In summary, our findings demonstrate that Tpm4.1 is essential to maintain cell-cell adhesions and to inhibit abnormal increases in cell migration and invasion, which are important to prevent invasion and metastasis of breast cancer cells. Tpm4.1 contributes to the regulation of Rac1 activity and localization of myosin IIB for maintenance of cell polarity. The mechanism by which Tpm4.1 regulates cell-cell adhesions and cell migration and invasion with Rac1 and myosin IIB remains to be determined. Elucidating the role of this newly identified tropomyosin isoform, Tpm4.1, to regulate the invasive behaviors of cancer cells will provide important insights into cancer invasion and metastasis.

## MATERIALS AND METHODS

### Cell culture and reagents

Untransformed breast epithelial cell line MCF10A and the breast cancer cell line MDA-MB-231 were obtained from ATCC. The parental MDA-MB-231 and its metastatic derivatives LM2, BoM2, and BrM2 were generously provided from Dr. Joan Massagué. MCF10A was cultured in DMEM/F-12 containing 5% horse serum, 1.05 mM CaCl2, 10 μg/ml insulin, 0.5 μg/ml hydrocortisone, 0.1 μg/ml cholera toxin, 20 ng/ml EGF, and 10 mM Hepes buffer. MDA-MB-231 cell lines were cultured in DMEM containing 10% FBS and 2 mM L-glutamine. Pharmacological reagents, NSC23766 (Sigma, SML0952), ML-7 (Enzo Life Sciences, BML-EI197), Y27632 (Enzo Life Sciences, ALX-270-333), were used to specifically inhibit Rac1, MLCK and ROCK, respectively.

### Immunoblot analysis

Cells were lysed with Laemmli sample buffer containing 125 mM Tris-HCl (pH 6.8), 4% SDS, 20% glycerol, β-mercaptoethanol, a protease inhibitor cocktail (Sigma), and a phosphatase inhibitor, PhosSTOP (Roche). The sample lysates were separated by SDS-PAGE and transferred to nitrocellulose membrane. The membrane was blocked with 5% skim milk and incubated with primary antibodies, TM311 (Sigma, T2780), E-cadherin (BD Transduction Laboratories, 610181), β-catenin (BD Transduction Laboratories, 610154), phospho-paxillin (Tyr 118) (Cell Signaling, 2541), paxillin (Santa Cruz, sc-5574), phospho-MLC2 (Ser19) (Cell Signaling, 3671), MLC2 (Santa Cruz, sc-15370), Myosin heavy chain IIA (Covance, PRB-440P), Myosin heavy chain IIB (Covance, PRB-445P), and β-actin (Sigma, A5441). The information of TM311, LC24, α/9d, and δ/9d (a synonym for WD4/9d) is described previously [15]. Primary antibodies were detected with HRP-conjugated goat anti-mouse IgG (Jackson Immuno Research, 115-035-003) and goat anti-rabbit IgG (Jackson Immuno Research, 111-035-003). The intensity of bands was measured using Sigma ScanPro4.

### RT-PCR analysis

Total mRNA was isolated from cells using RiboEx (GeneAll) according to the manufacturer's protocol. RT-PCR was performed using PrimeScript One-step RT–PCR Kit Ver. 2 (TaKaRa) according to the manufacturer's protocol. The following primer was used for Tpm4.1 (5′-GGAGCAGGCGGAGGCGGATA-3′).

### RNA interference and plasmids

For silencing Tpm4.1, three siRNA sequences were designed (#1, GAAAGCCGCUGAGGACAAG; #2, UGGAGCUCACGGAGAAGAA; #3, GGAUAAGA AAGCCGCUGAG) and used as a pool of all the siRNAs to reduce artificial silence. Tpm4.2 siRNA sequence is ACGCAAGAUCCAGGCCCU. NMHC IIA (1100442) and NMHC IIB (1100318) were purchased from Bioneer. Transfection of 25–50 nM siRNA was performed with Lipofectamine RNAiMAX (Invitrogen) twice in each experiment. The human Tpm4.1 cDNA plasmid (clone ID OVARC1001731) was purchased from NITE Biological Resource Center and cloned into the pCGN vector. The Tpm4.1 cDNA used for rescue experiments is generated by silent mutations of the siRNA-targeted sequences in the wild type human Tpm4.1 cDNA. For transient expression of cDNA plasmids, MCF10A cells were transfected with X-tremeGENE HP DNA transfection reagent (Roche) and MDA-MB-231 cells were transfected with Lipofectamine 3000 (Invitrogen) according to the manufacturer's protocol.

### Wound healing assay

After transfection of siRNA, MCF10A cells were replated on plate and cultured until formation of a uniform monolayer. Then scratch was made by a pipette tip. The migrated area was measured using ImageJ software.

### Boyden chamber migration and invasion assay

Monolayer-cultured MCF10A cells transfected with siRNA were incubated with serum free medium. Then the cells were detached from plate and seeded on naked PET membrane (Corning, 353097) or Matrigel-coated PET membrane with 8μm pores (Corning, 354483) at 1 × 10^5^ cells/well. After 48hr, the upper side of membrane was cleaned and cells on the bottom of membrane were fixed with 4% formaldehyde and stained with DAPI. The migrated cells were counted from five images/well and the mean cell number was obtained from two sets of each experiment.

### Immunofluorescence and live cell imaging

MCF10A cells were replated on coverslips coated with 10 μg/ml laminin (Sigma, L2020) after siRNA transfection. The cells were co-permeabilized with 0.1% Triton-X-100 in formaldehyde and fixed with 4% formaldehyde. MDA-MB-231 were replated on uncoated glass coverslips after siRNA transfection. The cells were fixed with 4% formaldehyde and permeabilized with 0.1% Triton-X-100 in PBS. The fixed cells were stained with the same primary antibodies used for immunoblot and vinculin (Sigma, V9131). The secondary staining was performed with Alexa 488- or 594-conjugated anti-mouse or anti-rabbit antibodies. To detect actin filaments Anti-stain 555 or Alexa 647-conjugated phalloidin was used. The nuclei were detected with DAPI. Images were obtained by Zeiss observer Z1 with Apotome2. For live cell imaging videos, the Differential Interference Contrast (DIC) images of MCF10A cells on a laminin-coated glass were recorded at 5-min intervals using Zeiss observer Z1.

### Spheroid formation and invasion assays

After transfection of siRNA, cells were detached and resuspended. The cells were distributed into ultra-low attachment round bottom 96 well plate (Costar, 7007) at 10,000 cells/well of MCF10A or 15,000 cells/well of MDA-MB-231 with 2.5% Matrigel. The plate was centrifuged at 1,000 g for 10 min. After incubation, the formed spheroids were embedded in 1.5 mg/ml type I collagen (PureCol, Advanced BioMatrix, 5005), which was neutralized using a 8:1 PureCol and 1M NaOH in ultra-low attachment flat bottom 96 well plate (Corning, 3474). The images were obtained by phase-contrast microscope (Eclipse TS100, Nikon).

### Rac1 activity assay

After transfection of siRNA, MCF10A cells were replated on culture plate and grown for the experiment. The level of GTP-bound Rac1 was analyzed using G-LISA Rac1 activation assay kit (Cytoskeleton, BK126) according to the manufacturer's protocol.

### Statistical analysis

The significance of experimental results was determined by Student's *t*-test using Microsoft Excel.

## SUPPLEMENTARY MATERIALS FIGURES AND TABLES








