# Supplementary material for: Loss of Tpm4.1 leads to disruption of cell-cell adhesions and invasive behavior in breast epithelial cells via increased Rac1 signaling
Source: Oncotarget. 2017 Apr 4;8(20):33544–59. doi: 10.18632/oncotarget.16825 (PMC5464889; doi:10.18632/oncotarget.16825)
Supplement: Supplementary file 1 [file oncotarget-08-33544-s001.pdf]

Loss of Tpm4.1 leads to disruption of cell-cell adhesions and invasive behavior in breast epithelial cells via increased Rac1 signaling

Supplementary Materials

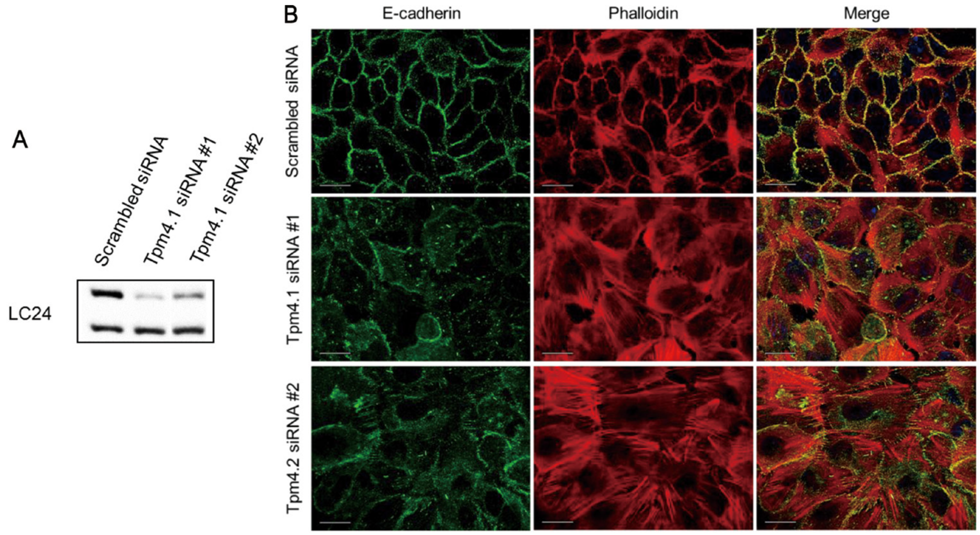

Supplementary Figure 1: (**A–B**) MCF10A cells were transfected with one of Tpm4.1 siRNAs. (A) Expression of Tpm4.1 was analyzed by immunoblot after siRNA transfection. (B) MCF10A cells with the indicated siRNAs were stained with the E-cadherin antibody and phalloidin to detect F-actin. The seeded cells on uncoated glass coverslips were transfected with siRNA and fixed. Scale bars, 20 µm.

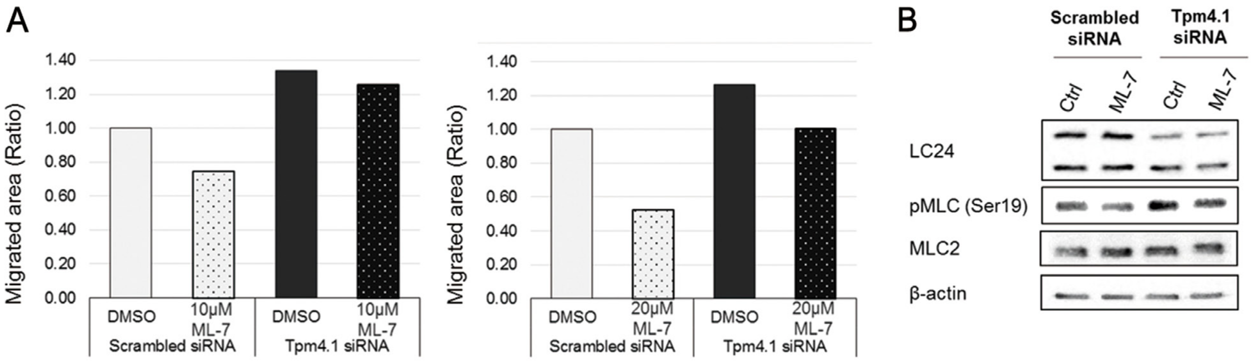

Supplementary Figure 2: (**A**) siRNA-transfected MCF10A cells were treated with the indicated concentration of ML-7 and migratory ability was analyzed by wound healing assay. (**B**) Phosphorylation of myosin light chains was analyzed by immunoblot in the same set used for wound healing assay with 10 µM ML-7.

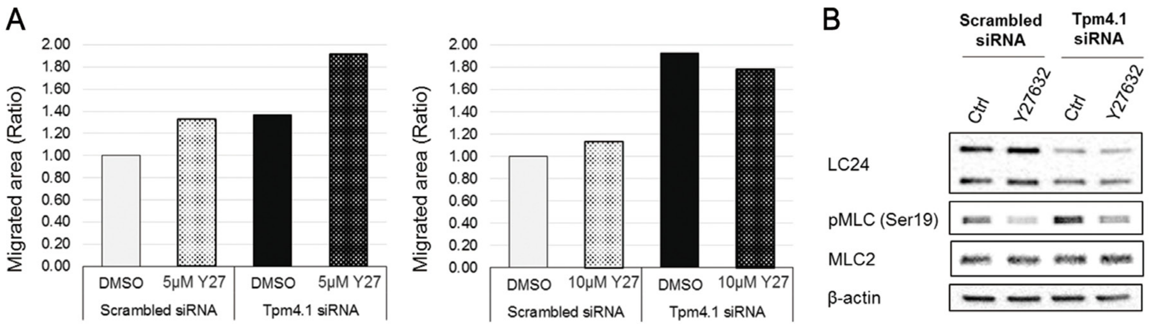

Supplementary Figure 3: (**A**) siRNA-transfected MCF10A cells were treated with the indicated concentration of Y27632 and migratory ability was analyzed by wound healing assay. (**B**) Phosphorylation of myosin light chains was analyzed by immunoblot in the same set used for wound healing assay with 5 μM Y27632.

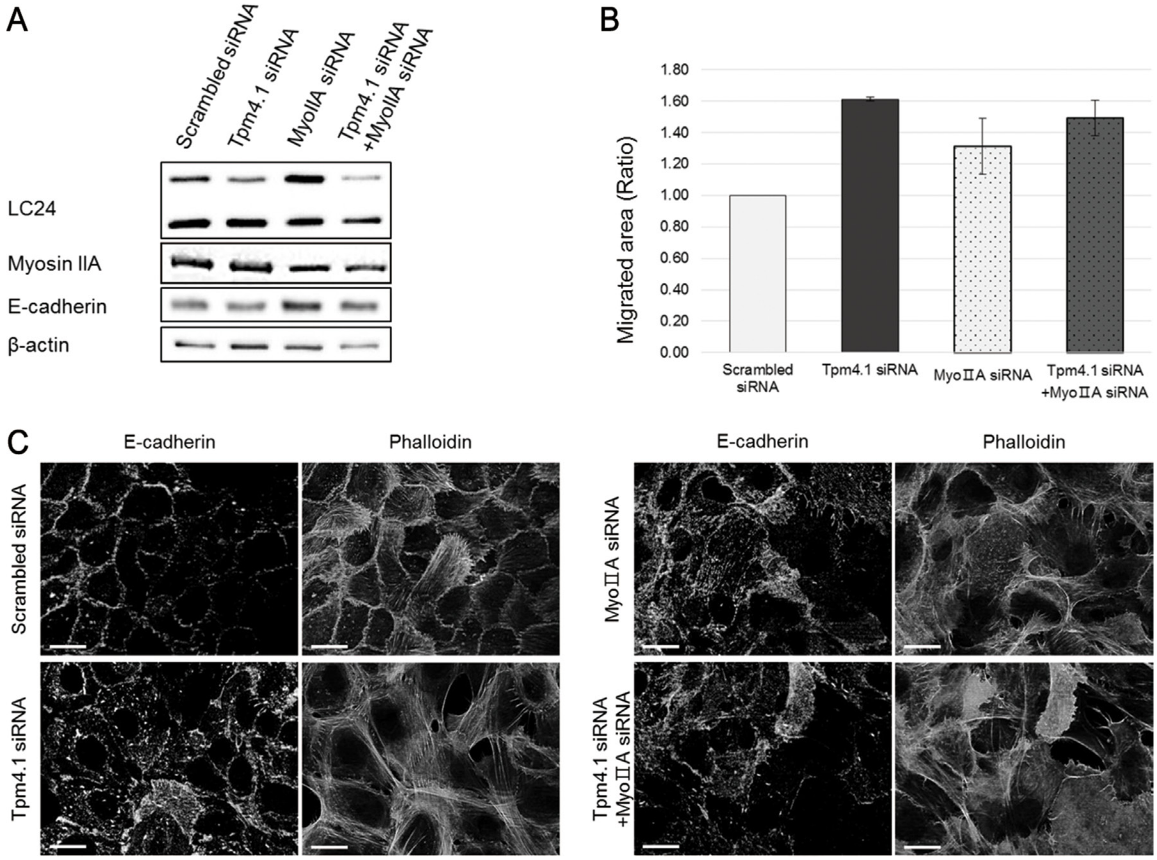

Supplementary Figure 4: (**A**) Immunoblot analysis after siRNA transfection. (**B**) Migratory ability of MCF10A cells with the indicated siRNAs was analyzed by wound healing assay. The graph was from two independent experiments. (**C**) MCF10A cells with the indicated siRNAs were stained with the E-cadherin antibody and phalloidin. Scale bars, 20 μm.

Supplementary Movie 1: Time-lapse video of MCF10A cells treated with scrambled siRNA shown in figure 2B.
See Supplementary_Movie_1

Supplementary Movie 2: Time-lapse video of MCF10A cells treated with Tpm4.1 siRNA shown in figure 2B.
See Supplementary_Movie_2

Supplementary Movie 3: Time-lapse video of MCF10A cells treated with Tpm4.2 siRNA shown in figure 2B.
See Supplementary_Movie_3
